# The Synergistic Effect of Conditional *Pten* Loss and Oncogenic *K-ras* Mutation on Endometrial Cancer Development Occurs via Decreased Progesterone Receptor Action

**DOI:** 10.1155/2010/139087

**Published:** 2009-10-27

**Authors:** Tae Hoon Kim, Jinrong Wang, Kevin Y. Lee, Heather L. Franco, Russell R. Broaddus, John P. Lydon, Jae-Wook Jeong, Francesco J. DeMayo

**Affiliations:** ^1^Department of Molecular and Cellular Biology, Baylor College of Medicine, Houston, TX 77030, USA; ^2^Joslin Diabetes Center, Harvard Medical School, Boston, MA 02215, USA; ^3^Department of Pathology, University of Texas, M.D. Anderson Cancer Center, Houston, TX 77030, USA

## Abstract

Endometrial cancer is the most common gynecological cancer. Estrogen-dependent endometrioid carcinoma is the most common type of endometrial cancer, and alterations in the expression of *PTEN* and *K-ras* have been associated with this disease. To study the roles of *Pten* and *K-ras* in endometrial cancer, we generated *Pten* ablation and oncogenic *K-ras* mutation in progesterone receptor positive cells (*PR* ^*cre*/+^ *Pten* ^*f*/*f*^ *K*-*ras* ^*G*12*D*^). Double mutant mice dramatically accelerated the development of endometrial cancer compared to a single mutation of either gene. Histological analysis showed that all of the 1-month old double mutant female mice developed endometrial cancer with myometrial invasion. The expression of PR was downregulated in double mutant mice compared to a single mutation of either gene which resulted in decreased suppression of estrogen signaling. Therefore, these results suggest a synergistic effect of dysregulation of the *Pten* and *K-ras* signaling pathways during endometrial tumorigenesis.

## 1. Introduction

Endometrial cancer is the most common type of gynecological cancer in the United States with approximately 40 100 cases diagnosed and about 7470 deaths from the disease each year [1]. Therefore, there is great interest in identifying novel ways to treat and prevent this disease. Estrogen-dependent endometrioid carcinoma is the most common type of endometrial cancer. An increased incidence of endometrial cancer has been found in association with prolonged, unopposed estrogen (E2) exposure [2, 3] and alterations in the expression of the tumor suppressor gene, *PTEN*, as well as the oncogenes, *β*-catenin and Ras [4, 5]. Currently, progesterone (P4) therapy is used as a means to prevent the development of endometrial cancer associated with unopposed E2 as a means to block E2 actions [6]. 

The uterus consists of heterogeneous cell types that undergo dynamic changes in response to the ovarian steroid hormones E2 and P4 to support embryo development and implantation. E2 stimulates the proliferation of epithelial cells in the mouse uterus [7, 8]. In contrast, P4 is inhibitory to this E2-mediated proliferation of the luminal and glandular epithelial cells. However, P4, alone or in conjunction with E2, leads to uterine stromal cell proliferation [8–10]. The ability of the ovarian steroid hormones to regulate uterine cell proliferation depends upon the ability of hormonal stimulation to regulate growth factor communication networks between the uterine stroma and epithelium. For instance, P4 attenuates E2-stimulated uterine epithelial cell proliferation by regulating uterine stromal cell gene expression [11]. An imbalance caused by increased E2 action and/or decreased P4 action can result in abnormal endometrial proliferation and endometrial adenocarcinoma. Therefore, elucidating the molecular mechanisms by which the steroid hormones control uterine physiology is important to understanding the pathology of these diseases.

Two common mutations that occur in endometrial cancer are in the tumor suppressor *Pten *and the oncogene *K-ras * [4, 5].* K-ras* encodes a guanine nucleotide binding protein of 21 kDa that has a central role in the regulation of cell growth and differentiation by transducing signals from activated transmembrane receptors. It has been shown to be mutated in 10%–30% of endometrial cancers [4]. These mutations have been found in all grades of endometrioid carcinoma and have been reported in complex atypical hyperplasia, suggesting a relatively early role for *K-ras* mutations in endometrial tumorigenesis [12]. *PTEN* (phosphatase and tensin homologue deleted from chromosome 10) is one of the most frequently mutated tumor suppressor genes in human cancers [13, 14]. *PTEN* is completely lost or mutated in >50% of primary endometrioid endometrial cancer [15] and in at least 20% of endometrial hyperplasias, the precancerous lesions of the endometrium [15, 16]. Thus, loss of *PTEN* is a very early event in the multistep process leading to endometrioid endometrial cancer [16, 17]. Phosphoinositide 3-kinases (PI3K) regulates a number of cellular functions through the activation of Akt [18]. *PTEN* acts as a negative regulator of PI3K signaling [19]. Previously, loss of *Pten* (either as a heterozygote or by uterine specific ablation) has been shown to induce endometrial cancer in mice highlighting its important role in cancer development [20, 21]. This mutation and subsequent Akt activation resulted in the activation of ER*α*-dependent pathways that play an important role in the tumorigenesis of endometrial cancer [21]. Interestingly, the *PTEN*/PI3K/AKT signaling pathway can also be activated by E2 suggesting a complex interaction between these two signaling pathways [22]. 

In this study, we utilized conditional *Pten* ablation and oncogenic *K-ras* mutation in the uteri of mice to demonstrate a synergistic effect of dysregulation of the *Pten* and *K-ras* signaling pathways during endometrial tumorigenesis. *Pten* ablation and oncogenic *K-ras* mutation dramatically accelerated the development of endometrial cancer compared to single mutation of either gene. Thus, these results demonstrate the importance of *Pten *and* K-ras *regulation in the tumorigenesis of endometrial cancer.

## 2. Materials and Methods

### 2.1. Animals

 mice were maintained in the designated animal care facility at Baylor College of Medicine according to the institutional guidelines for the care and use of laboratory animals. *PR* ^*Cre*/+^ mice were previously generated [23]. The *Pten* ^*f*/*f*^ were acquired from Dr. Hong Wu (University of California, Los Angeles, Los Angeles, CA) [24]. The lox-stop-Lox *Kras* ^G12D^ mice were acquired from Dr. Tyler Jacks (MIT, Cambridge, MA) [25]. Mice of various genotypes were sacrificed at 2 and 4 weeks of age. At the time of dissection, uterine tissues were placed in the appropriate fixative or flash frozen and stored at −80°C. For the genotyping of the oncogenic *K-ras* mutation, total RNA was isolated from uterus according to, Qiagen minieasy kit protocol. cDNA was generated from RNA samples by reverse transcription, followed by PCR amplification using primers *K-ras*1S, 5′-GCCATTTCGGACCCGGAGCGA and *K-ras*1A, 5′-CCTACCAGGACCATAGGCACATC. RNA expression of wild-type *K-ras* and mutant *K-ras* *G*12*D* was determined by digestion of 5 *μ*L of the reverse transcription-PCR products with HindIII for 1 hour at 37°C. The restriction products were resolved in a 2% agarose gel. The mutant *K-ras *G12D allele contains a HindIII restriction site engineered in exon 1, which is absent in the wild-type allele. Therefore, digestion of the 488-bp products generates 300-bp and 148-bp restriction fragments in the mutant but not in the wild-type PCR product.

### 2.2. Western Blot Analysis

Samples containing 15 *μ*g of proteins were applied to SDS-PAGE. The separated proteins were transferred onto a polyvinylidene difluoride membrane (Millipore Corp., Bedford, MA). Membranes were blocked overnight with 0.5% casein (wt/vol) in PBS with 0.1% Tween 20 (vol/vol) (Sigma-Aldrich, St. Louis, MO) and probed with anti-Akt (Santa Cruz Biotechnology, Inc., Santa Cruz, CA), anti-phospho-Akt (Cell Signaling Technology, Inc., Danvers, MA), anti-PR (DAKO Corp., Capinteria, CA), or anti-ER*α* (DAKO Corp., Capinteria, CA) antibodies. Immunoreactivity was visualized by incubation with a horseradish peroxidase-linked secondary antibody and treatment with ECL reagents. To control for loading, the membrane was stripped and probed with anti-actin (Santa Cruz Biotechnology, Inc., Santa Cruz, CA) and developed again.

### 2.3. Immunohistochemistry and TUNEL Assay

Uterine sections from paraffin-embedded tissue were cut at 5 *μ*m and mounted on silane-coated slides, deparafinized, and rehydrated in a graded alcohol series. Sections were preincubated with 10% normal serum in PBS (pH 7.5) and then incubated with anti-PR antibody (DAKO Corp., Capinteria, CA) or anti-ER*α* (DAKO Corp., Capinteria, CA) in 10% normal serum in PBS (pH 7.5). On the following day, sections were washed in PBS and incubated with a secondary antibody (5 *μ*L/mL; Vector Laboratories, Burlingame, CA) for 1 hour at room temperature. Immunoreacitivity was detected using the Vectastain Elite ABC kit (Vector Laboratories, Burlingame, CA). The TUNEL assay was performed according to manufacturer's instructions using the Roche In Situ Cell Death Detection Kit, Fluorescein (Roche, Boulder, CO).

### 2.4. RNA Isolation and Quantitative Real-Time RT-PCR

Total RNA was extracted from uterine tissues using the Qiagen RNeasy total RNA isolation kit (Valencia, CA). Quantitative real-time RT-PCR analysis was conducted on isolated RNA. Expression levels of *Ltf, Clca3,* and *C3* were measured by real-time RT-PCR TaqMan analysis (Applied Biosystems, Foster City, CA). cDNA was made from 1 *μ*g of total RNA using random hexamers and M-MLV Reverse Transcriptase (Invitrogen Corp., Carlsbad, CA). RT-PCR was performed using RT-PCR Universal Master Mix reagent (Applied Biosystems, Foster City, CA). All real-time RT-PCR results were normalized against 18S RNA using ABI rRNA control reagents. Statistical analyses were performed using one-way ANOVA followed by Tukey's post hoc multiple range test with the Instat package from GraphPad (San Diego, CA, USA).

## 3. Results

### 3.1. Generation of Pten Ablation and Oncogenic K-Ras Mutation in the Murine Uterus

**Figure 1:**
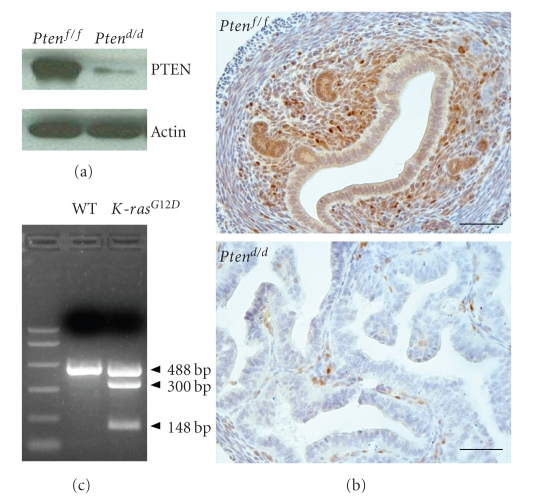
Analysis of conditionally ablated *Pten* and oncogenic* K-ras *mutation in the murine uterus. (a) Western blot analysis of *PTEN* in whole uterine extracts from *Pten^f/f^* and *Pten^d/d^* mice. (b) Immunohistochemical analysis for *PTEN* in *Pten^f/f^* and *Pten^d/d^* mice uteri. Four-week-old* Pten^f/f^* and *Pten^d/d^* mice were used for Western blot analysis and immunohistochemical analysis. These experiments demonstrate that *Pten* is conditionally ablated in the uteri of these mice. Scale bar: 50 *μ*m. (c) PR-cre mediated recombination of *K-ras* ^*G*12*D*^ in uterus. The mutant *K-ras G12D* allele contains a HindIII restriction site engineered in exon 1, which is absent in the wild-type allele. Therefore, digestion of the 488-bp products generates 300-bp and 148-bp restriction fragments in the mutant but not in the wild-type PCR product.

Homozygous *Pten* ^−/−^ mouse embryos die around E8.5 and heterozygous *Pten* ^+/−^ mice develop numerous pathologies and have decreased longevity [21, 26] making our ability to investigate the role of *Pten* in the mouse uterus severely limited. Likewise, constitutive activation of a *K-ras *mutation results in embryonic lethality [27]. In order to achieve ablation of *Pten* and activation of *K-ras* in the uterus, mice with floxed *Pten *(*Pten* ^*f*/*f*^) [24] and mice with loxP-Stop-loxP-*Kra* *s* ^*G*12*D*/+^ (LSL-*K-ras* ^*G*12*D*/+^) [25] were bred to the *P* *R* ^*Cre*^ mouse [23]. Using this mouse with Cre recombinase inserted into the progesterone receptor (PR) locus, floxed genes are edited in PR expressing cells including all compartments of the mouse uterus. This model was previously used to ablate *Pten* in the uterus resulting in endometrial adenocarcinoma [20]. Therefore, in order to effectively investigate the effects of the *Pten* and *K-ras* signaling pathways in endometrial cancer, mice with uterine *Pten *ablation (*PR^cre/+^Pten* ^*f/f*^; *Pten* ^*d/d*^) and oncogenic *K-ras *mutation (*K-ras* ^*G*12*D*^) were generated and mated to generate double mutant mice (*PR^cre/+^Pten^f/f^K-ras* ^*G*12*D*^; *Pten^d/d^K-ras* ^*G*12*D*^) [23–25]. Ablation of *Pten* in the *Pten^d/d^* mice was assayed by Western blot and immunohistochemical analysis (Figures 1(a) and 1(b)). *PTEN* protein was expressed in the endometrium of *Pten^f/f^* mice. However, the level of *PTEN* protein was significantly decreased in the uteri of *Pten^d/d^* mice demonstrating efficient ablation of *Pten*. To confirm the oncogenic *K-ras* mutation in the uterus*, *the PCR products encompassing the* K-ras *mutation were digested with HindIII (see Section 2). The mutant *K-ras* G12D allele contains a HindIII restriction site engineered in exon 1, which is absent in the wild-type allele. Therefore, digestion of the 488-bp products generates 300-bp and 148-bp restriction fragments in the mutant but not in the wild-type PCR product. Analysis of the HindIII digestion revealed the presence of the 300-bp and 148-bp fragments in the *K-ras* ^*G*12*D*^ but not in the wild type uteri (Figure 1(c)). These results suggest that PR-cre efficiently generated uterine *Pten* ablation and oncogenic *K-ras* mutation.

### 3.2. Development of Vaginal Papillomas in Mice with the Oncogenic *K-ras* Mutation in PR-Expressing Cells

**Figure 2:**
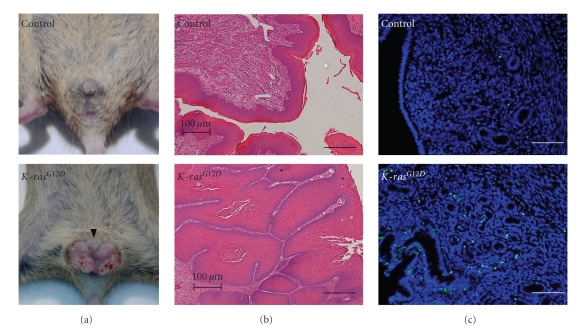
Development of vaginal papiloma and increased apoptosis in *K-ras* ^*G*12*D*^ mice. (a) The development of vaginal papiloma. Control and *K-ras* ^*G*12*D*^ mice at 2 months of age. (b) H&E staining of vagina of control and *K-ras* ^*G*12*D*^ mice. The histology of these lesions revealed an abnormal vaginal architecture in the *K-ras* ^*G*12*D*^ mice compared to controls. (c) TUNEL assay in the uterus of control and *K-ras* ^*G*12*D*^ mice at 2 months of age. The number of apoptotic cells was significantly increased in epithelial cells of *K-ras* ^*G*12*D*^ uteri compared to controls. Scale bar: 100 *μ*m.

Introduction of the oncogenic *K-ras *mutation in all PR-positive cells resulted in the development of vaginal papillomas. Lesions were present at the vaginal opening of *K-ras* ^*G*12*D*^ mice as early as 2 months of age but were never observed in control mice (Figure 2(a)). Examination of the histology of these lesions revealed an abnormal vaginal architecture in the *K-ras* ^*G*12*D*^ mice compared to controls (Figure 2(b)). The vaginal epithelium exhibited increased keratinization accompanied by disorganization of the vaginal lumen. In addition, there was a decrease in the vaginal stroma. Interestingly, these lesions remained benign, never developing into a cancerous lesion. *K-ras* ^*G*12*D*^ mice did not show any pathological phenotype in the uterus. Proliferation was not affected in the *K-ras* mutant mice; however, there was increased apoptosis in the *K-ras* ^*G*12*D*^ uteri compared to controls (Figure 2(c)).

### 3.3. Development of Endometrial Cancer in Mice with Pten Ablation and the Oncogenic *K-ras *Mutation in PR-Expressing Cells

**Figure 3:**
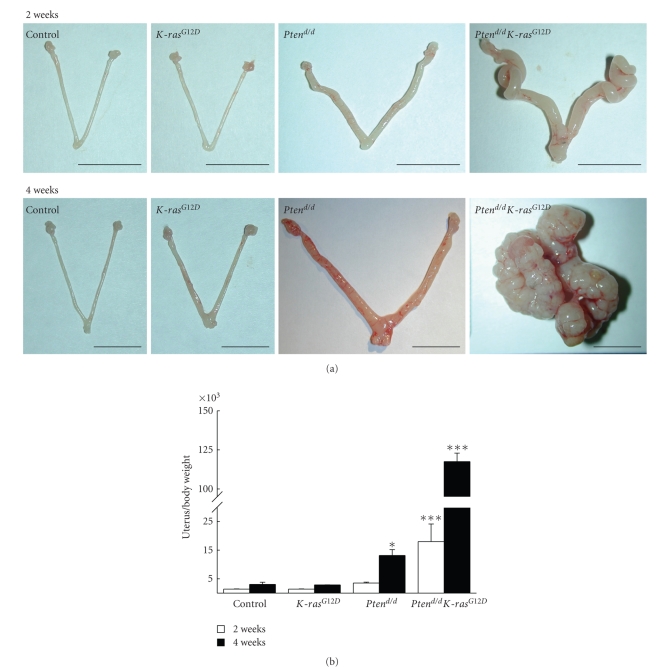
Development of endometrial cancer in mice with* Pten *ablation and oncogenic* K-ras *mutation. (a) Gross anatomy of control, *K-ras* ^*G*12*D*^, *Pten^d/d^*, and *Pten^d/d^K-ras* ^*G*12*D*^ uteri at 2 and 4 weeks of age. Scale bar: 1 cm (b) The ratio of uterine weight to body weight in control, *K-ras* ^*G*12*D*^, *Pten^d/d^*, and *Pten^d/d^K-ras* ^*G*12*D*^ mice at 2 and 4 weeks of age. Uterine weight was determined for females 2 and 4 weeks old. Increased uterine weight was observed for *Pten^d/d^K-ras* ^*G*12*D*^ mice after 2 weeks of age. *, *P* < .05; ***, *P* < .001, one-way ANOVA followed by Tukey's post hoc multiple range test.

To investigate the impact of *Pten *and *K-ras *signaling on endometrial cancer development and progression, control, *Pten^d/d^*, *K-ras* ^*G*12*D*^, and *Pten^d/d^K-ras* ^*G*12*D*^ mice were sacrificed, excised uteri were weighed, and morphology was examined at gross and histological levels. *Pten^d/d^K-ras* ^*G*12*D*^ mice showed a significant increase in uterine weight at 2 weeks of age compared to control, *Pten^d/d^*, and *K-ras* ^*G*12*D*^ mice (Figures 3(a) and 3(b)). *Pten^d/d^* and *Pten^d/d^K-ras* ^*G*12*D*^ mice showed a significant increase in uterine wet weight compared to control mice at 4 weeks of age. The uterine weight of *Pten^d/d^ K-ras* ^*G*12*D*^ mice was significantly increased compared to other mice including *Pten^d/d^* mice at 4 weeks of age (Figures 3(a) and 3(b)). The uterine weight of *K-ras* ^*G*12*D*^ mice did not change compared to control mice at either timepoint. 

**Figure 4:**
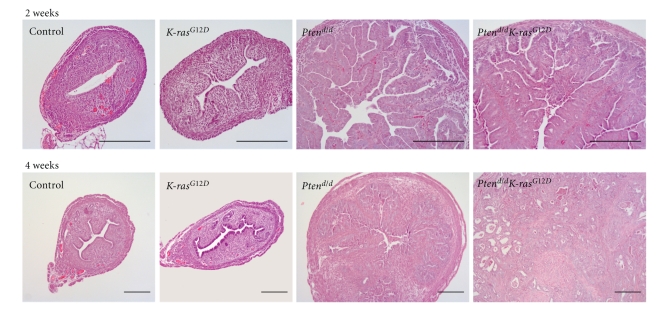
Histology of uteri from mice with* Pten *ablation and oncogenic* K-ras *mutation. H&E staining of control, *K-ras* ^*G*12*D*^, *Pten^d/d^*, and *Pten^d/d^K-ras* ^*G*12*D*^ mice at 2 and 4 weeks of age. Endometrial cancer was induced in the uteri of *Pten^d/d^K-ras* ^*G*12*D*^ mice, but not in other mice at 4 weeks of ages. Scale bar: 200 *μ*m.

Histological analysis of the uteri showed an increase in the number of endometrial glands and in the gland/stroma ratio in the uteri of 2-week-old *Pten^d/d^* and *Pten^d/d^K-ras* ^*G*12*D*^ mice (Figure 4); however, the myometrium was not enlarged. These histological changes demonstrate that the uteri of 2 week old *Pten^d/d^* and *Pten^d/d^K-ras* ^*G*12*D*^ mice display endometrial hyperplasia, a predisposing factor to endometrial adenocarcinoma in humans. Thus, even though the uterine weight of the *Pten^d/d^K-ras* ^*G*12*D*^ mice was increased compared to the *Pten^d/d^* mice, the uteri of *Pten^d/d^* and *Pten^d/d^K-ras* ^*G*12*D*^ mice exhibit a similar hyperplastic phenotype at 2 weeks of age. 

Interestingly, all of the *Pten^d/d^K-ras* ^*G*12*D*^ mice developed invasive endometrioid-type endometrial adenocarcinoma by 4 weeks of age. The neoplastic endometrial glands in the *Pten^d/d^K-ras* ^*G*12*D*^ mice invaded through the uterine muscle wall and invaded adjacent structures such as the colon, pancreas, and skeletal muscle (Figure 3(a)). The *Pten^d/d^* mice displayed endometrial hyperplasia at 4 weeks of age (Figure 4). Although the *Pten^d/d^* mice have been previously shown to develop endometrial hyperplasia and endometrial cancer [20], they did not develop invasive endometrial cancer within 4 weeks of age. Thus, histological analysis showed that *Pten* ablation in conjunction with the oncogenic *K-ras* mutation dramatically accelerated the development of endometrial cancer compared to single ablation of either gene (Figure 4). These results suggest that *Pten* ablation in addition to the oncogenic *K-ras* mutation dramatically accelerated the development of endometrial cancer compared to single mutation of either gene.

### 3.4. Down-regulation of P4 Signaling in Mice with Pten Ablation and the Oncogenic *K-ras *Mutation

**Figure 5:**
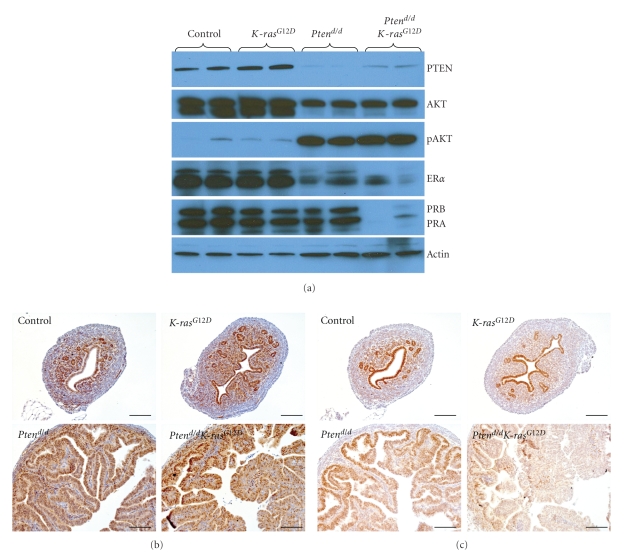
The decrease of PR in the 2-week-old *Pten^d/d^K-ras* ^*G*12*D*^ mice. (a) Western blot analysis of *PTEN*, AKT, phosphor-AKT, ER*α*, and PR in 2 week old control, *K-ras* ^*G*12*D*^, *Pten^d/d^*, and *Pten^d/d^K-ras* ^*G*12*D*^ mice. (b)-(c) Immunohistochemical analysis of ER*α* (b) and PR (c) in uteri of control, *K-ras* ^*G*12*D*^, *Pten^d/d^*, and *Pten^d/d^K-ras* ^*G*12*D*^ mice. Immunohistochemical analysis of PR shows that it is decreased in the *Pten^d/d^K-ras* ^*G*12*D*^ uteri compared to other mice. Scale bar: 100 *μ*m.

Ablation of *Pten* resulted in increased activation of AKT as expected (Figure 5(a)). To determine if the hyperplastic phenotype observed was due to altered ovarian steroid hormone signaling, we examined the expression of ER*α* and PR in control, *K-ras* ^*G*12*D*^, *Pten^d/d^*, and* Pten^d/d^K-ras* ^*G*12*D*^ uteri at 2 weeks of age using Western blot analysis. The expression of ER*α* was decreased in *Pten^d/d^* and* Pten^d/d^K-ras* ^*G*12*D*^ uteri compared to control and *K-ras* ^*G*12*D*^ uteri (Figure 5(a)). Interestingly, the level of PR, both the PR-A and PR-B isoforms, was decreased only in the* Pten^d/d^ K-ras* ^*G*12*D*^ uteri compared to control, *K-ras* ^*G*12*D*^, and* Pten^d/d^* uteri at 2 weeks of age (Figure 5(a)). 

To analyze the spatial expression of ER*α* and PR, we performed immunohistochemical analysis in control, *K-ras* ^*G*12*D*^, *Pten^d/d^*, and* Pten^d/d^K-ras* ^*G*12*D*^ uteri at 2 weeks of age. The expression of ER*α* was decreased in the endometrial stroma of *Pten^d/d^ K-ras* ^*G*12*D*^ compared to *Pten^d/d^* mice. However, the level of ER*α* was increased in the epithelium of *Pten^d/d^K-ras* ^*G*12*D*^ compared to *Pten^d/d^* mice. These immunohistochemical results suggest that the decreased level of ER*α *in the whole uterus was due primarily to decreased ER*α* expression in the endometrial stroma (Figure 5(b)). The spatial expression of PR was altered in the *Pten^d/d^* uteri compared to control and *K-ras* ^*G*12*D*^ mice. Instead of uniform expression throughout the endometrial epithelium, PR was localized to the distal regions of the epithelium in the *Pten^d/d^* uteri (Figure 5(c)). However, this strong PR expression was almost lost in the *Pten^d/d^K-ras* ^*G*12*D*^ mice.

**Figure 6:**
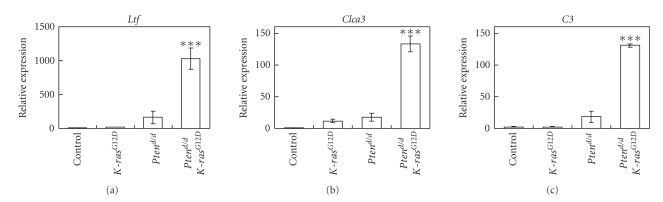
The increase of ER*α* signaling in the 2-week-old *Pten^d/d^K-ras* ^*G*12*D*^ mice. Real-time RT-PCR analysis of *Ltf, Clca3,* and *C3* was performed in uteri of control, *K-ras* ^*G*12*D*^, *Pten^d/d^*, and *Pten^d/d^K-ras* ^*G*12*D*^ mice at 2 weeks of age. The results represent the mean ± SEM of three independent RNA sets. ***, *P* < .001, one-way ANOVA followed by Tukey's post hoc multiple range test.

We next investigated the molecular impact of *Pten* ablation and the oncogenic *K-ras* mutation on the expression of ER target genes (*Ltf, Clca3, *and* C3*) in control, *K-ras* ^*G*12*D*^, *Pten^d/d^*, and* Pten^d/d^K-ras* ^*G*12*D*^ uteri at 2 weeks of age. *Ltf *and* C3* were increased in the *Pten^d/d^* mice as compared to control and *K-ras* ^*G*12*D*^ mice (Figure 6). *Ltf, Clca3, *and* C3 *were significantly increased in the* Pten^d/d^K-ras* ^*G*12*D*^ mice as compared to *Pten^d/d^* mice. Since P4 attenuates E2 regulation of proliferation and gene expression by regulating the expression of a yet to be identified paracrine signal from the stromal cells to the epithelial cells, the regulation of the expression of ER*α* and PR in the endometrial stroma and epithelium by *Pten *ablation and the oncogenic* K-ras *mutation is critical for the expression of ER target genes and the tumorigenesis of endometrial cancer.

## 4. Discussion

Endometrial cancer is the most common gynecological cancer and has been shown to be associated with mutations in the tumor suppressor *Pten *and the oncogene *K-ras *among others [4]. Previous mouse model studies in the skin, ovary, and lung suggest that these mutations exert cooperative or antagonistic effects on tumorigenesis depending upon their interactions with tissue-specific factors [28–30]. Dinulescu et al. generated *Pten* loss and oncogenic *K-ras* mutations in ovarian surface epithelium using adenoviral vector delivery of Cre recombinase [28]. These mice developed endometriosis and endometrioid ovarian adenocarcinoma. *K-ras* mutations occur in a very small percentage of human cases of ovarian cancer but the mutation is important for the development of ovarian cancer [31]. However, whether *Pten* loss and oncogenic *K-ras* mutations interact to promote or inhibit the development of endometrial cancer has not yet been defined. In order to study the role of *Pten* and *K-ras* in the development of endometrial cancer, we generated mice in which *Pten* was ablated and *K-ras* was activated in the reproductive tract using the *PR^Cre^* mouse model [23–25]. 

*PTEN* is completely lost or mutated in >50% of primary endometrioid endometrial cancer [15] and in at least 20% of endometrial hyperplasias, the precancerous lesions of the endometrium [15, 16]. Thus, loss of *PTEN* is a very early event in the multistep process leading to endometrioid endometrial cancer. *Pten* ^+/−^ and mice with *Pten *conditionally ablated in the uterus (*Pten^d/d^*) develop endometrioid endometrial adenocarcinoma [20, 32]. This mutation and subsequent Akt activation results in the activation of ER*α*-dependent pathways that play an important role in the tumorigenesis of endometrial cancer [21]. Introduction of the oncogenic *K-ras *mutation into the *Pten^d/d^* mice accelerated the tumorigenesis of endometrial cancer as compared to *Pten* ablation. The neoplastic endometrial glands in the double mutant mice invaded through the uterine muscle wall and invaded adjacent structures such as the colon, pancreas, and skeletal muscle. 

The *K-ras *mutation alone was not sufficient enough to exert a pathological phenotype in the uterus even though mutations in *K-ras* have been consistently identified in 10%–30% of endometrial cancers [4]. Interestingly, *K-ras* ^*G*12*D*^ mice exhibited abnormal vaginal architecture due to increased keratinization in the vaginal epithelium resulting in vaginal papilloma. The development of vaginal papillomas confounds its impact on the tumorigenesis of endometrial cancer. Thus, we have only examined these mice up to 3 months of age without evidence of any hyperplasia or pathological phenotype in the uterus. To determine why endometrial cancer failed to develop in the *K-ras* ^*G*12*D*^ uteri, we examined proliferation and apoptosis in these mice. Proliferation was normal; however, apoptosis was significantly increased in the epithelial cells of *K-ras* ^*G*12*D*^ uteri compared to control mice as shown by the TUNEL assay (Figure 2(c)) and immunohistochemistry of cleaved caspase 3 (data not shown). These results suggest that activation in *K-ras* is not sufficient for the development of hyperplasia or endometrial cancer due to increased apoptosis of the endometrial epithelial cells. 

Mutations in the *β*-catenin gene have been found in approximately 15%–20% of endometrioid carcinomas, with nuclear accumulation of the protein found in 38% of cases [33]. Subsets of endometrial carcinomas, especially those with nuclear translocation of *β*-catenin [34], are associated with squamous morule differentiation. Some of the tumors in *Pten^d/d^* mice exhibited squamous differentiation, which can also be observed in human endometrial cancers while we did not observed squamous differentiation in the tumors from* Pten^d/d^K-ras* ^*G*12*D*^ mice (data not shown). Nuclear translocation of *β*-catenin was not observed in the *Pten^d/d^* mice and *Pten^d/d^K-ras* ^*G*12*D*^ mice (data not shown). It indicates that the histopathology of the endometrial cancer lesions in the mice is very similar to human endometrial cancer but does not totally resemble human endometrial cancer which is mainly composed of glandular components with squamous differentiation. 

ER and PR are usually found in high concentration in endometrial hyperplasia and endometrioid carcinomas of low grade and stage. However, the level of ER and PR diminishes with increases in stage and grade [35, 36]. The level of total ER*α* was decreased in *Pten^d/d^* and* Pten^d/d^K-ras* ^*G*12*D*^ uteri compared to control and *K-ras* ^*G*12*D*^ uteri using Western analysis (Figure 5(a)). The amount of epithelial cells in *Pten^d/d^* and* Pten^d/d^K-ras* ^*G*12*D*^ uteri is much higher than control and *K-ras* ^*G*12*D*^ mice because of the hyperplasia phenotype. The amount of epithelial cells was different in the 4 different groups of the Western analysis because we have not purified epithelial cells and normalized to epithelial marker proteins. Therefore, we performed immunohistochemical analysis to determine these possible compartmental differences. The results demonstrated that the expression of ER*α* was decreased in the endometrial stroma but was increased in the endometrial epithelium of *Pten^d/d^K-ras* ^*G*12*D*^ compared to *Pten^d/d^* mice. These results support that epithelial ER*α* is important for the tumorigenesis of endometrial cancer [4].

E2 induces cell proliferation in the luminal and glandular epithelium. In the uterine luminal epithelium, E2 inhibits GSK3*β* action by the stimulation of a protein kinase B-(AKT)-mediated inhibitory phosphorylation of Ser9. AKT is in turn regulated through activation of phosphoinositide 3-kinase [37]. P4 inhibits this pathway by blocking AKT phosphorylation and, thus, the inactivation of GSK3*β* with the resultant loss of nuclear cyclin D1 [37]. PR status is considered an independent prognostic factor for endometrial cancer patients [38, 39]. PR exists as two isoforms, PR-A and PR-B, and reduced expression of either one or both of the PR isoforms has been observed in a great majority of endometrial cancers, compared with hyperplastic or normal endometrium [40]. Loss of PR in human endometrioid endometrial carcinoma results in more aggressive biological characteristics which play important roles in the prognosis and recurrence of the disease [40–42]. We observed reduced expression of both PR isoforms in *Pten^d/d^K-ras* ^*G*12*D*^ uteri, but not in *Pten^d/d^* or other control uteri. Thus, this loss of PR in the double mutant mice may be the reason for the accelerated tumorigenesis of the *Pten^d/d^K-ras* ^*G*12*D*^ mice as compared to other mice including the *Pten^d/d^* mice. 

Our results demonstrate that the synergistic effect of conditional* Pten *loss and oncogenic* K-ras *mutation on endometrial cancer development occurs via decreased expression of PR. This study has established an endometrial cancer mouse model which replicates common characteristics of the human disease. Using this mouse model, further studies can be undertaken to investigate the genetic and molecular events involved in the transition from normal to hyperplastic/neoplastic endometrium. In summary, these results greatly contribute to the understanding of the molecular mechanism of tumorigenesis and to the development of therapeutic approaches for endometrial cancer.

## References

[1] Jemal A, Siegel R, Ward E (2008). Cancer statistics, 2008. *CA: Cancer Journal for Clinicians*.

[2] Jick SS, Walker AM, Jick H (1993). Estrogens, progesterone, and endometrial cancer. *Epidemiology*.

[3] Ziel HK, Finkle WD (1975). Increased risk of endometrial carcinoma among users of conjugated estrogens. *New England Journal of Medicine*.

[4] Di Cristofano A, Ellenson LH (2007). Endometrial carcinoma. *Annual Review of Pathology*.

[5] Hecht JL, Mutter GL (2006). Molecular and pathologic aspects of endometrial carcinogenesis. *Journal of Clinical Oncology*.

[6] Jick SS (1993). Combined estrogen and progesterone use and endometrial cancer. *Epidemiology*.

[7] Martin L, Finn CA, Trinder G (1973). Hypertrophy and hyperplasia in the mouse uterus after oestrogen treatment: an autoradiographic study. *Journal of Endocrinology*.

[8] Huet-Hudson YM, Andrews GK, Dey SK (1989). Cell type-specific localization of c-myc protein in the mouse uterus: modulation by steroid hormones and analysis of the periimplantation period. *Endocrinology*.

[9] Paria BC, Huet-Hudson YM, Dey SK (1993). Blastocyst's state of activity determines the ‘window’ of implantation in the receptive mouse uterus. *Proceedings of the National Academy of Sciences of the United States of America*.

[10] Martin L, Das RM, Finn CA (1973). The inhibition by progesterone of uterine epithelial proliferation in the mouse. *Journal of Endocrinology*.

[11] Kurita T, Lee K-J, Cooke PS, Lydon JP, Cunha GR (2000). Paracrine regulation of epithelial progesterone receptor and lactoferrin by progesterone in the mouse uterus. *Biology of Reproduction*.

[12] Esteller M, Garcia A, Martinez-Palones JM, Xercavins J, Reventos J (1997). The clinicopathological significance of K-RAS point mutation and gene amplification in endometrial cancer. *European Journal of Cancer*.

[13] Steck PA, Pershouse MA, Jasser SA (1997). Identification of a candidate tumour suppressor gene, MMAC1, at chromosome 10q23.3 that is mutated in multiple advanced cancers. *Nature Genetics*.

[14] Li D-M, Sun H (1997). TEP1, encoded by a candidate tumor suppressor locus, is a novel protein tyrosine phosphatase regulated by transforming growth factor *β*. *Cancer Research*.

[15] Sun H, Enomoto T, Fujita M (2001). Mutational analysis of the PTEN Gene in endometrial carcinoma and hyperplasia. *American Journal of Clinical Pathology*.

[16] Levine RL, Cargile CB, Blazes MS, van Rees B, Kurman RJ, Ellenson LH (1998). PTEN mutations and microsatellite instability in complex atypical hyperplasia, a precursor lesion to uterine endometrioid carcinoma. *Cancer Research*.

[17] Maxwell GL, Risinger JI, Gumbs C (1998). Mutation of the PTEN tumor suppressor gene in endometrial hyperplasias. *Cancer Research*.

[18] Vivanco I, Sawyers CL (2002). The phosphatidylinositol 3-kinase-AKT pathway in human cancer. *Nature Reviews Cancer*.

[19] Jiang B-H, Liu L-Z (2008). PI3K/PTEN signaling in tumorigenesis and angiogenesis. *Biochimica et Biophysica Acta*.

[20] Daikoku T, Hirota Y, Tranguch S (2008). Conditional loss of uterine Pten unfailingly and rapidly induces endometrial cancer in mice. *Cancer Research*.

[21] Vilgelm A, Lian Z, Wang H (2006). Akt-mediated phosphorylation and activation of estrogen receptor *α* is required for endometrial neoplastic transformation in Pten +/− mice. *Cancer Research*.

[22] Chambliss KL, Yuhanna IS, Anderson RGW, Mendelsohn ME, Shaul PW (2002). Er*β* has nongenomic action in caveolae. *Molecular Endocrinology*.

[23] Soyal SM, Mukherjee A, Lee KYS (2005). Cre-mediated recombination in cell lineages that express the progesterone receptor. *Genesis*.

[24] Lesche R, Groszer M, Gao J (2002). Cre/loxP-mediated inactivation of the murine Pten tumor suppressor gene. *Genesis*.

[25] Johnson L, Mercer K, Greenbaum D (2001). Somatic activation of the K-ras oncogene causes early onset lung cancer in mice. *Nature*.

[26] Di Cristofano A, Pesce B, Cordon-Cardo C, Pandolfi PP (1998). Pten is essential for embryonic development and tumour suppression. *Nature Genetics*.

[27] Tuveson DA, Shaw AT, Willis NA (2004). Endogenous oncogenic K-rasG12D stimulates proliferation and widespread neoplastic and developmental defects. *Cancer Cell*.

[28] Dinulescu DM, Ince TA, Quade BJ, Shafer SA, Crowley D, Jacks T (2005). Role of K-ras and Pten in the development of mouse models of endometriosis and endometrioid ovarian cancer. *Nature Medicine*.

[29] Iwanaga K, Yang Y, Raso MG (2008). Pten inactivation accelerates oncogenic K-ras-initiated tumorigenesis in a mouse model of lung cancer. *Cancer Research*.

[30] Mao J-H, To MD, Perez-Losada J, Wu D, Del Rosario R, Balmain A (2004). Mutually exclusive mutations of the Pten and ras pathways in skin tumor progression. *Genes and Development*.

[31] Kurman RJ, Shih I-M (2008). Pathogenesis of ovarian cancer: lessons from morphology and molecular biology and their clinical implications. *International Journal of Gynecological Pathology*.

[32] Lian Z, De Luca P, Di Cristofano A (2006). Gene expression analysis reveals a signature of estrogen receptor activation upon loss of Pten in a mouse model of endometrial cancer. *Journal of Cellular Physiology*.

[33] Fukuchi T, Sakamoto M, Tsuda H, Maruyama K, Nozawa S, Hirohashi S (1998). *β*-catenin mutation in carcinoma of the uterine endometrium. *Cancer Research*.

[34] Saegusa M, Hashimura M, Kuwata T, Hamano M, Okayasu I (2005). Upregulation of TCF4 expression as a transcriptional target of *β*-catenin/p300 complexes during trans-differentiation of endometrial carcinoma cells. *Laboratory Investigation*.

[35] Emons G, Fleckenstein G, Hinney B, Huschmand A, Heyl W (2000). Hormonal interactions in endometrial cancer. *Endocrine-Related Cancer*.

[36] Kleine W, Maier T, Geyer H, Pfleiderer A (1990). Estrogen and progesterone receptors in endometrial cancer and their prognostic relevance. *Gynecologic Oncology*.

[37] Chen B, Pan H, Zhu L, Deng Y, Pollard JW (2005). Progesterone inhibits the estrogen-induced phosphoinositide 3-kinase → AKT → GSK-3*β* → cyclin D1 → pRB pathway to block uterine epithelial cell proliferation. *Molecular Endocrinology*.

[38] Mohsin SK, Weiss H, Havighurst T (2004). Progesterone receptor by immunohistochemistry and clinical outcome in breast cancer: a validation study. *Modern Pathology*.

[39] Rose PG (1996). Endometrial carcinoma. *New England Journal of Medicine*.

[40] Arnett-Mansfield RL, deFazio A, Wain GV (2001). Relative expression of progesterone receptors A and B in endometrioid cancers of the endometrium. *Cancer Research*.

[41] Fukuda K, Mori M, Uchiyama M, Iwai K, Iwasaka T, Sugimori H (1998). Prognostic significance of progesterone receptor immunohistochemistry in endometrial carcinoma. *Gynecologic Oncology*.

[42] Ito K, Utsunomiya H, Yaegashi N, Sasano H (2007). Biological roles of estrogen and progesterone in human endometrial carcinoma—new developments in potential endocrine therapy for endometrial cancer. *Endocrine Journal*.

